# A case of bronchiolitis obliterans organizing pneumonia in an HIV-infected Korean patient successfully treated with clarithromycin

**DOI:** 10.1186/s12879-015-1025-6

**Published:** 2015-07-23

**Authors:** In Young Jung, Yong Duk Jeon, Mi-young Ahn, Eunkyong Goag, EunHye Lee, Hea Won Ahn, Jin Young Ahn, Nam Su Ku, June Myung Kim, Jun Yong Choi

**Affiliations:** Department of Internal Medicine, Yonsei University College of Medicine, 50 Yonsei-ro, Seodaemun-gu, Seoul, 120-752 South Korea; AIDS Research Institute, Yonsei University College of Medicine, Seoul, South Korea

**Keywords:** HIV, BOOP, clarithromycin

## Abstract

**Background:**

Bronchiolitis obliterans organizing pneumonia (BOOP) is a type of diffuse interstitial lung disease characterized by the pathology of fibroblastic plugs in the lumens of the respiratory bronchioles, alveolar ducts, and alveoli. The occurrence of BOOP in human immunodeficiency virus (HIV)-infected patients has rarely been described, and there have been no clinical case reports in Korea.

**Case presentation:**

A 24-year-old female who had been diagnosed with HIV ten years prior was admitted due to a 1-year history of cough and sputum production and a 3-day history of fever. She had poor adherence to anti-retroviral therapy (ART) due to gastrointestinal troubles. At the time of admission, her CD4 T-cell count was 5 cells/mm^3^. A high resolution computed tomography (CT) scan showed tiny centrilobular nodules with a tree-in-bud pattern in both lungs. Bacterial culture, *Pneumocystis jirovecii* polymerase chain reaction (PCR), *Aspergillus* galactomannan antigen (Ag) assay, and respiratory virus PCR were negative. Rapid chest x-ray improvement was seen after a 7-day treatment with anti-tuberculosis medication, ceftriaxone, and clarithromycin. Miliary tuberculosis seemed unlikely considering the rapid radiologic improvement and negative tuberculosis PCR results. Due to the unknown etiology, we performed video-assisted thoracoscopic surgery (VATS) to determine the cause of the diffuse lung infiltration. Pathologic findings were consistent with BOOP, while tissue acid-fast bacilli (AFB) stain and tuberculosis PCR results were negative. Tuberculosis medication and intravenous ceftriaxone were discontinued, while treatment with clarithromycin monotherapy was sustained. Five months after discharge, the patient was asymptomatic with a normal chest x-ray and as her adherence to ART improved, her CD4 T-cell count rose to 181 cells/mm^3^. Clarithromycin was discontinued at that time and the patient is currently receiving regular outpatient follow-up.

**Conclusion:**

This case suggests that macrolides are a potential treatment option in HIV-infected patients with mild BOOP. In cases that are otherwise unexplained or unresponsive to treatment, BOOP should be taken into consideration and surgical biopsy performed to confirm a diagnosis of BOOP.

## Background

Bronchiolitis obliterans organizing pneumonia (BOOP) is a type of diffuse interstitial lung disease characterized by the pathology of fibroblastic plugs in the lumens of the respiratory bronchioles, alveolar ducts, and alveoli [1]. Various lung diseases have been reported in patients infected with human immunodeficiency virus (HIV), but there are few reports of the occurrence of BOOP in HIV patients and prior to this study, there was no clinical case report in Korea [2]. Here, we describe a unique case of BOOP in a HIV-infected patient diagnosed by surgical lung biopsy and successfully treated with clarithromycin.

## Case presentation

**Fig. 1:**
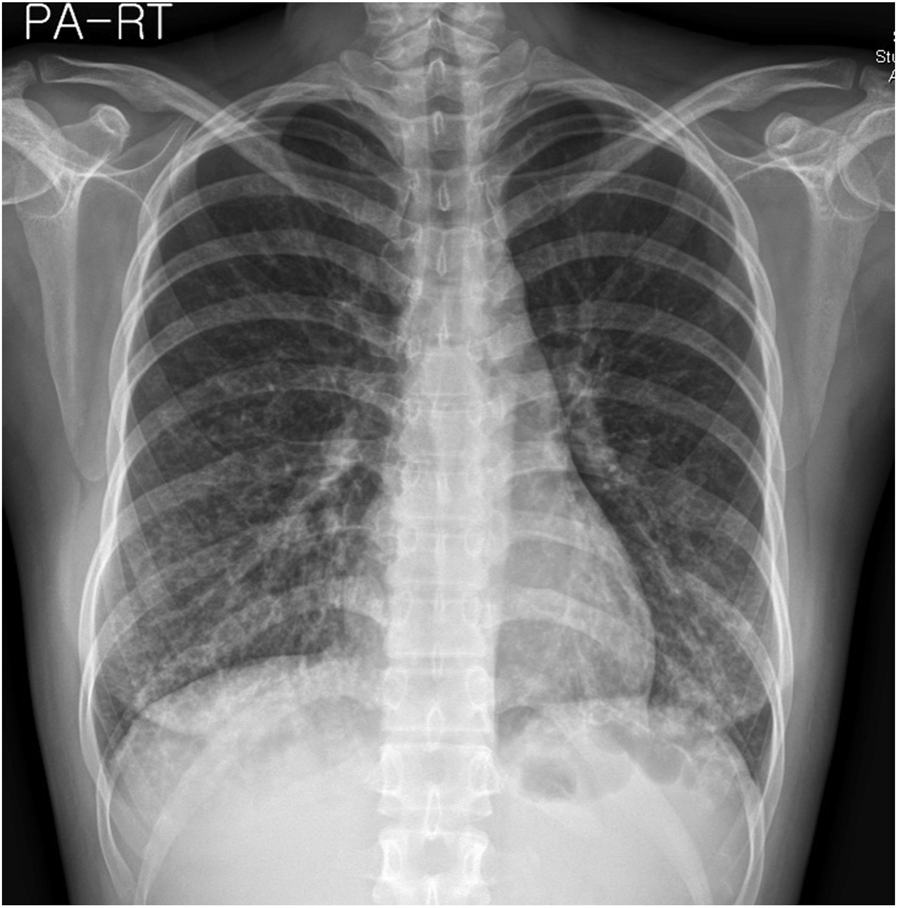
Chest radiography on admission. Diffuse bilateral reticular infiltrates in the bilateral lung fields were present

**Fig. 2:**
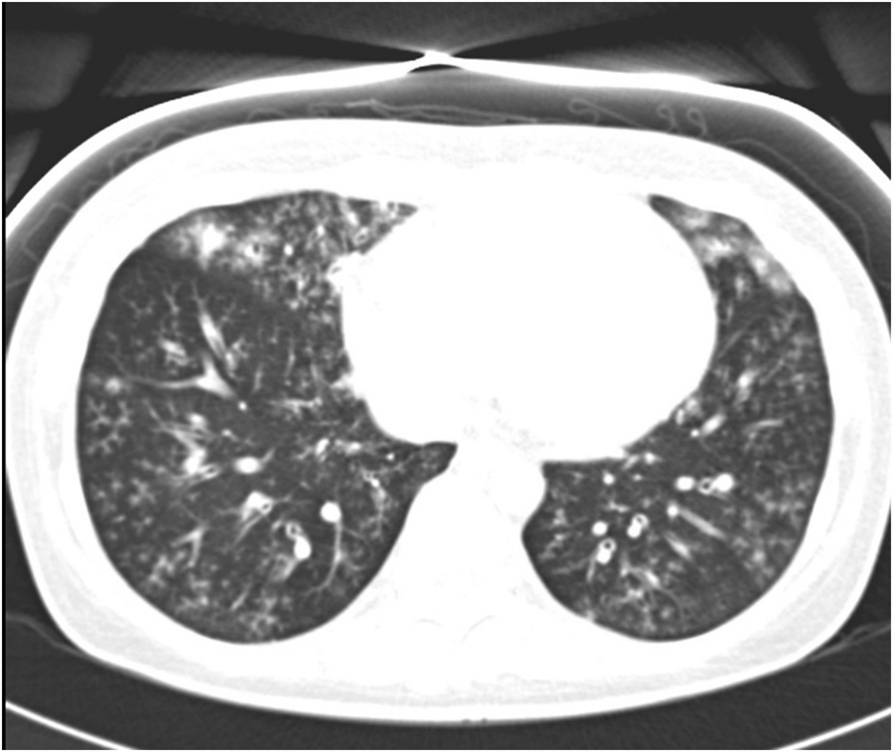
High resolution computed tomography (CT) scan showing tiny centrilobular nodules with a tree-in-bud pattern in both lungs

**Fig. 3:**
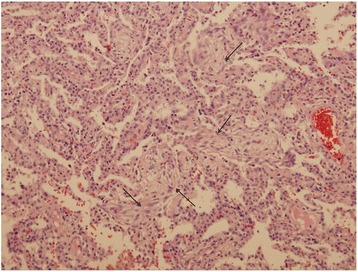
Microscopic examination of the lung tissue at x100 magnification after H&E staining. Patchy fibroblastic plugs in the bronchioles and alveolar ducts were observed, consistent with BOOP

**Fig. 4:**
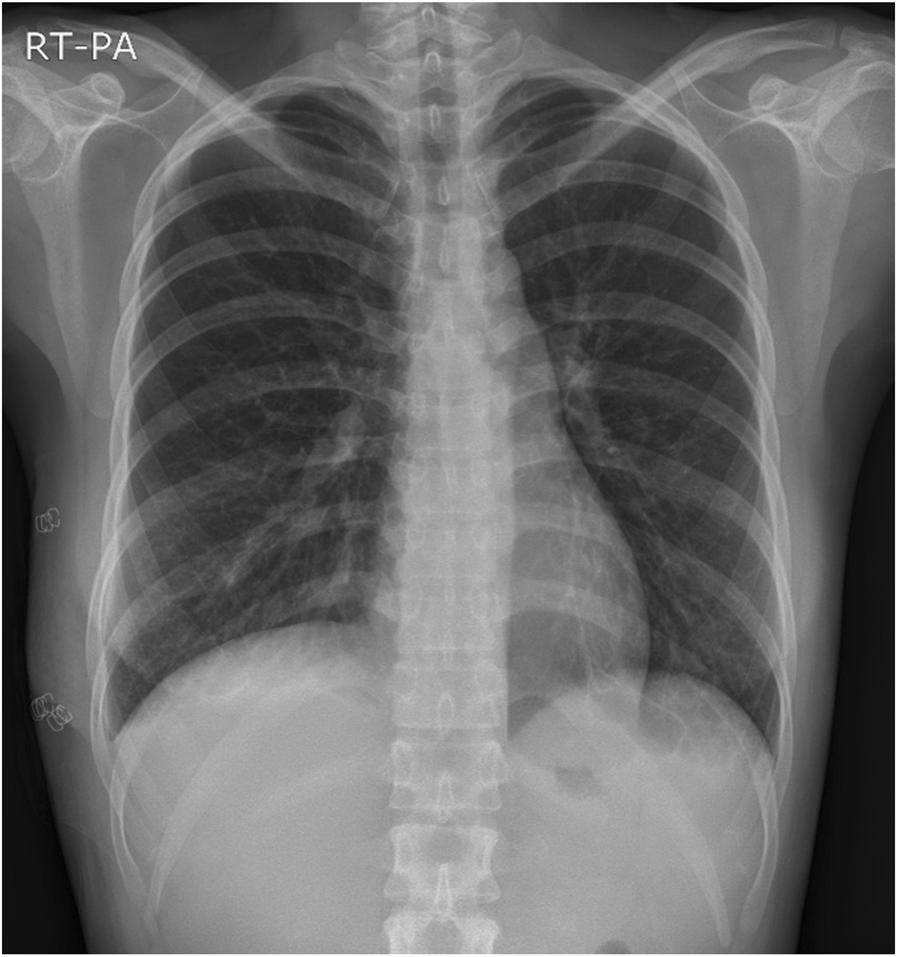
Chest X-ray on the 15^th^ day of admission. Note resolution of the diffuse bilateral reticular infiltrates

A 24-year-old female was admitted for a 1-year history of cough and sputum production and a 3-day history of fever. She had been diagnosed with HIV infection 10 years prior, and prescribed anti-retroviral therapy medications (ART; Rilpivirine 25 mg once/day, Lamivudine 300 mg/ Abacavir 600 mg once/day, Raltegravir 400 mg twice/day), but had poor adherence due to gastrointestinal troubles. Her CD4 T-cell count 3 months before admission was 15 cells/mm^3^, and her viral load had not been suppressed for several years. On admission, her temperature was 37 °C, respiratory rate was 20 breaths per minute, pulse was 108 beats per minute, and blood pressure was 102/74 mmHg. Auscultation of her lungs revealed coarse breath sounds with crackles in the bilateral lower lung fields. A complete blood cell count and blood chemistry on admission revealed a white blood cell count of 4,240/mm^3^ (neutrophils, 88.5 %; lymphocytes, 6.1 %), hemoglobin level of 13.0 g/dL, and platelet count of 66,000/mm^3^. Arterial blood gas analysis measured while breathing room air showed an arterial oxygen partial pressure (PaO_2_) of 74.2 mmHg, arterial carbon dioxide partial pressure (PaCO_2_) of 37.4 mmHg, and oxygen (O_2_) saturation of 95.3 %. Quantitative real-time polymerase chain reaction showed a HIV viral load of 851,000 copies/mL, and the patient’s CD4 T cell count was 5 cells/mm^3^. Chest radiography on admission revealed diffuse bilateral reticular infiltrates in bilateral lung fields (Fig. 1). A high resolution computed tomography (CT) scan showed tiny centrilobular nodules with a tree-in-bud pattern in both lungs (Fig. 2). To evaluate the possibility of community-acquired bacterial pneumonia, a urine *Streptococcus pneumoniae* antigen (Ag) test, sputum *Legionella pneumophila* polymerase chain reaction (PCR) test, and sputum and blood cultures were done. All results were negative. Viral pneumonia was also considered and PCR tests were performed to detect adenovirus, respiratory syncytial virus, influenza viruses A and B, coronavirus, and parainfluenza virus, but all results were negative. *Pneumocystis jirovecii* PCR, *Aspergillus* galactomannan Ag assay, and cryptococcal Ag test results were also negative. We considered cytomegalovirus (CMV) pneumonitis, and CMV quantitative PCR revealed 13,650 copies/mL. However, the patient’s clinical symptoms and chest x-ray improved without any treatment for CMV. Miliary tuberculosis was initially suspected, and anti-retroviral agents (elvitegravir 150 mg, cobicistat 288.5 mg, emtricitabine 200 mg, tenofovir 300 mg) and anti-tuberculosis medication (isoniazid 300 mg once/day, ethambutol 1200 mg once/day, pyrazinamide 1500 mg once/day, and rifabutin 300 mg 3 times/week) were administered. At the same time, ceftriaxone 2 g once daily and clarithromycin 500 mg twice daily were administered, as the clinicians were unable to rule out concurrent bacterial infection. On the 7th day of hospitalization, the patient’s chest x-ray showed rapid improvement. The patient did not present with any symptoms suggestive of inflammatory connective tissue diseases, such as arthralgia or dry eyes, and had no exposure history to irradiation or fumes, which are known to cause inflammatory injury to the lungs. Contrary to the clinicians’ initial suspicion of miliary tuberculosis, results of an interferon γ releasing assay (IGRA), sputum acid-fast bacilli (AFB) stain, and tuberculosis PCR were negative. Overall, the etiology remained unknown, so on the 8th day of admission, we performed video-assisted thoracoscopic surgery (VATS) to determine the cause of the patient’s diffuse lung infiltration. Microscopic examination showed patchy fibroblastic plugs in the bronchioles and alveolar ducts, consistent with BOOP, while tissue AFB stain and tuberculosis PCR tests were negative (Fig. 3). Anti-tuberculosis medications and ceftriaxone were discontinued, and oral clarithromycin 500 mg twice a day was continued. Follow-up chest radiography and clinical symptoms of cough and sputum production improved prior to discharge (Fig. 4). Five months after discharge, the patient was asymptomatic with a normal chest x-ray. Furthermore, as the patient’s adherence to ART improved, her CD4 T-cell count rose to 181 cells/mm^3^. Clarithromycin was discontinued at that time and the patient is currently receiving regular outpatient follow-up.

## **Discussio**n

BOOP is a diffuse interstitial lung disease that affects the small airways [1]. The precise incidence and prevalence of BOOP is unknown, though a study performed in Italy confirmed 78 cases of biopsy-proven BOOP in a 7 year period in a single center [3]. Clinical features of this disease include fever, mild dyspnea, and cough, similar to flu-like symptoms [4]. The pathogenesis of BOOP is thought to be due to epithelial injury followed by formation of fibrinoid inflammatory cell clusters and consequently intra-alveolar fibrosis [4]. The pathologic hallmark of BOOP is the presence of fibroblastic plugs in the lumens of the respiratory bronchioles, alveolar ducts, and alveoli. [5]. Due to these characteristic pathologic findings, biopsy for diagnosis of BOOP is essential [4, 5]. Transbronchial lung biopsy may be performed, but video-assisted thoracoscopic lung biopsy is the preferred technique because it is more likely to provide an adequate amount of tissue [4, 5]. Because BOOP occurs as a result of an inflammatory response to lung injury, a good treatment response is seen with broad-spectrum anti-inflammatory agents, such as corticosteroids and immune-modulatory macrolides [6]. A few case reports have described treatment of BOOP in non-HIV-infected patients with macrolide antibiotics, but there have been no studies of their use in HIV-positive patients [7]. High dose oral glucocorticoid therapy of 0.75-1 mg/kg per day is suggested for initial treatment of patients with severe symptoms [1]. There are no established guidelines regarding duration of treatment, though a total of 1 year is often recommended due to frequent relapses in cases in which therapy was discontinued earlier [4]. Immune-modulatory macrolides treat mild diseases such as BOOP by inhibiting pro-inflammatory cytokine production [8, 9].

There is a reasonable chance that BOOP in a HIV-infected patient may be underdiagnosed, considering that areas of high HIV prevalence are usually resource-limited and lung biopsies are not readily performed in these places. Until now, concurrent BOOP and HIV infection has been reported in only 11 patients. Diagnoses were made via transbronchial or thoracoscopic biopsy, and a rapid treatment response was seen with the use of corticosteroids. However, in one case, fatal pulmonary aspergillosis occurred, and CMV retinitis and esophagitis occurred in two cases. These complications imply that prolonged use of corticosteroids in HIV-infected BOOP patients may result in fatal clinical outcomes secondary to their immunocompromised state [2]. Both HIV and non-HIV infected patients respond well to glucocorticoid therapy; however, prolonged use of steroids in patients with concomitant HIV and BOOP can increase the risk of opportunistic infections [10]. While there are many known side-effects associated with long-term use of corticosteroids, macrolides have been shown to be effective and relatively safe to use on a long-term basis [6]. In previous case reports (Allen et al., Liote et al., Laguna Del Estal et al. and Santio et al.), HIV infected patients with BOOP presented with a low CD4 cell count or a low CD4/CD8 ratio, suggesting that severe immune deficiency may be needed for BOOP to develop in this scenario [11]. When treating HIV-infected patients who have low immune status, physicians should consider a diagnosis of BOOP in patients unresponsive to treatment for more common conditions, such as *Pneumocystis jirovecii* pneumonia, or when the etiology is unknown [12].

The limitations of this case report are as follows. The patient’s CD4 T-cell count improved significantly (from 5 to 181 cells/mm^3^) from the time of diagnosis of BOOP until the end of macrolide treatment. This improvement in immune status may have provided protection from other opportunistic infections. Empirical treatment with ceftriaxone may have improved the clinical course, and there is also the chance that the BOOP resolved spontaneously. Our evaluation of the patient’s response to clarithromycin treatment may not have been accurate for the above reasons.

## Conclusions

In summary, this case suggests that macrolides are a potential treatment option in HIV-infected patients with mild BOOP. In cases that are otherwise unexplained or unresponsive to treatment, BOOP should be taken into consideration and surgical biopsy performed to confirm a diagnosis of BOOP.

## Consent

Written informed consent was obtained from the patient for publication of this case report. A copy of the written consent is available for review by the Editor of this journal.
